# Analysis of Menaquinone-7 Content and Impurities in Oil and Non-Oil Dietary Supplements

**DOI:** 10.3390/molecules23051056

**Published:** 2018-05-01

**Authors:** Arkadiusz Szterk, Katarzyna Bus, Adam Zmysłowski, Karol Ofiara

**Affiliations:** Department of Spectrometric Methods, National Medicines Institute, 30/34 Chełmska, 00-725 Warsaw, Poland; k.bus@nil.gov.pl (K.B.); a.zmyslowski@nil.gov.pl (A.Z.); k.ofiara@nil.gov.pl (K.O.)

**Keywords:** dietary supplements, menaquinone, *cis*-isomers, *trans*-isomers, vitamin K_2_

## Abstract

Rapid, global technological development has caused the food industry to use concentrated food component sources like dietary supplements ever more commonly as part of the human diet. This study analysed the menaquinone-7 (MK-7) content of dietary supplements in oil capsule and hard tablet forms. A novel method for separating and measuring geometric isomers of MK-7 in dietary supplements was developed and validated. Eleven different isomers of *cis*/*trans-* menaquinone-7 were identified. Identification of *cis*/*trans* isomers was performed by combination of HPLC, UPLC and HRMS-QTOF detection, whereas their quantities were determined by DAD detection. The content of menaquinone impurities was ascertained, including *cis*/*trans-* menaquinone-6 isomers (5.5–16.9 µg per tablet/capsule) and *cis*/*trans*-menaquinone-7 isomers (70.9–218.7 µg tablet/capsule), which were most likely formed during the chemical synthesis of the menaquinone-7. The all-*trans* MK-7 content was lower than the isomeric form and often lower than what the labels declared. A new method of quantification, developed and validated for menaquinones in oil capsules, provided on average 90% recovery and a limit of quantification (LOQ) of approximately 1 µg mL^−1^.

## 1. Introduction

Rapid, worldwide technological development has brought about significant changes in the food industry consisting, among others, of people ingesting concentrated sources of food components as dietary supplements. These kinds of supplements are commonly available and constitute an ever-larger part of the human diet. The chemical composition and chemical transformations of dietary supplement ingredients are a very important and new area in the chemical research of foods, which is currently developing very dynamically. Analysing the market of dietary supplements, one discovers that vitamin preparations constitute a significant part of the market. Recently, vitamin MK-7, sold in the form of soft capsules containing different vegetable oils, as well as hard tablets, have become very popular.

**Figure 1:**
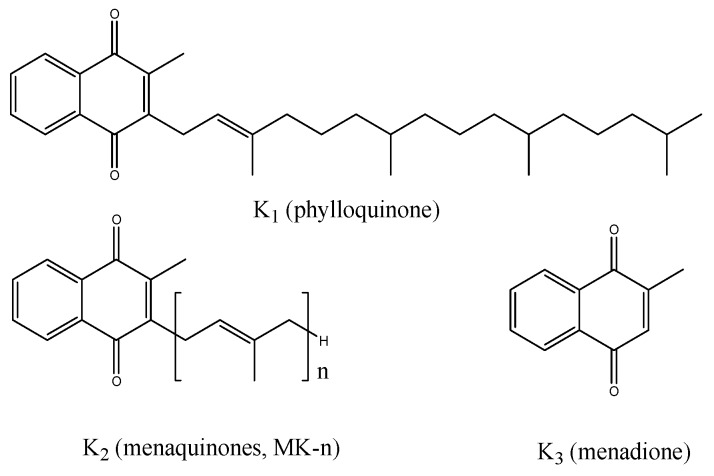
Chemical structure of vitamin K.

Vitamin K is a fat-soluble vitamin, and this name is applied to several different chemical substances containing the 2-methyl-1,4-naphthoquinone group with different lateral hydrocarbon chains in the C_3_ position [1]. There are two different forms of vitamin K in nature: vitamin K_1_ (phylloquinone or phytonadione), consisting of a long phytyl lateral chain and vitamin K_2_ (menaquinones), a feature of which is a long polyprenyl lateral chain [2] which were presented on Figure 1 [2].

Vitamin K exhibits biological activity due to the presence of the naphthoquinone ring. The reduced form of vitamin K is a cofactor for γ-glutamylcarboxylase, an enzyme involved in the post-translational modification of proteins by converting glutamic acid residues (Glu) to γ-carboxy-glutamic acid residues (Gla). The presence of Gla in proteins induces an affinity for calcium ions and determines their biological activity [3,4,5,6]. The Gla-protein family is involved in processes such as the regulating blood coagulation pathway, preventing calcification of blood vessels and increasing bone mineralization. Vitamin K_1_ is a cofactor of γ-glutamylcarboxylase that modifies hepatic blood coagulation factors. Vitamin K_2_ has a higher affinity for extrahepatic γ-glutamylcarboxylase, modifying proteins such as osteocalcin, matrix Gla-protein or growth arrest specific gene 6 protein [7,8,9]. Vitamin K forms differ not only in target tissues but also in biological activity, as long-chain menaquinines show greater activity compared to short-chain ones due to the longer half-life in the blood [4,10,11].

The main food sources of vitamin K are green plants and algae. High vitamin K_1_ concentrations are found in green vegetables (e.g., broccoli, iceberg lettuce, and spinach) and vegetable oils (e.g., soya oil and olive oil) [12]. Vitamin K_2_ is present in low concentrations in animal products such as eggs and meat, and in high concentrations (up to 1200 μg per 100 g) in fermented products such as natto, cheese, curdled milk and sauerkraut [11,12,13,14]. Based on the toxicological report of EFSA [15] the MK-7 vitamin intake for female adults is estimated at approximately 30 µg day^−1^, and, for male adults, approximately 50 µg day^−1^. Vitamin K_2_, in comparison with vitamin K_1_, is found in a few food products_;_ thus its daily and even long-term supply is limited [16,17]. Therefore, a good method for ensuring adequate vitamin K_2_ intake may be the dietary supplements commonly available on the global market.

Under the current law, the content of vitamins and minerals in the daily dose of dietary supplements recommended by the producer should take into account the upper safe intake levels, determined on the basis of scientific data. The reference daily intake of vitamin K for adult is equal to 75 µg. This value was developed based on the demand for phylloquinone, but taking into consideration lack of specific guidelines and recommendations for daily intake of menaquinones, most producers apply this value for dietary supplements containing MK-7. There are also no guidelines for the chemical quality of dietary supplements, which is significant in the case of MK-7. Menaquinones can be obtained from natural sources, mainly natto [18] or by chemical synthesis, which is much more economical [19,20,21,22]. Vitamin K_2_ preparations obtained from natto contain 100% *trans* MK-n, and the MK-7 form constitutes more than 95% of the menaquinones [16,18,23,24]. There are no reports in the literature pertaining to the contents of the *cis*/*trans* forms in natural MK-7 preparations. *Cis*/*trans* menaquinone isomer contents have been obtained for chemically synthesised preparations [19,20,21,22]. The research conducted by Lowenthal and Rivera [25] demonstrated that the *cis* forms of vitamin K exhibit merely 1% of the biological activity of the *trans* forms. The results of this study were also confirmed by other researchers [26,27,28]. Depending on the methods used for synthesis/biosynthesis and purification of the post-reaction mixture, the final preparation may contain both the *cis* and *trans* forms of MK-7 as well as other forms, particularly MK-6. The form MK-6 is the penultimate stage of the synthesis/biosynthesis process and is the most likely not to undergo a complete reaction to the MK-7 form, remaining in the final product [22]. The presence of different forms of vitamin K_2_, including *cis*/*trans* isomers is very important taking into consideration the biological properties of the vitamin preparations, because the impact on humans is not known.

The objective of this study was to research two kinds of MK-7 dietary supplements (oil capsules and hard tablets) for the presence of the *trans* form, the *cis/trans* form, and impurities, mainly the *cis/trans* form of MK-6, which may be present in supplements containing the synthetic form of this vitamin. A previously developed, novel analytical method, extremely selective in terms of *cis*/*trans* MK-7 isomers, was used. The second objective of this study was to develop a method for preparing oil samples for the quantification of the *cis/trans* MK-7 isomers. The final objective was to verify whether the MK-7 content declared on the packaging of both types of supplements was accurate.

## 2. Materials and Methods

### 2.1. Research Material

The research material was eight different dietary supplements of MK-7, four in the form of hard tablets and four in the form of soft gelatin capsules, in which the vitamin was dissolved in different vegetable oils. The supplements were purchased at 10 different pharmacies, and, for each supplement, *n* = 10. All the supplements in the form of a soft capsule were purchased in Poland. One of the hard tablet supplements was from Sweden, one was from Poland, and two were from Great Britain. All the researched supplements had labels declaring the MK-7 content or the mixture of MK-7 and vitamin D_3_.

### 2.2. Chemicals

Tetrahydrofuran (THF) for analysis, EMPARTA^®^ ACS (LiChrosolv, MERCK, Darmstadt, Germany); methanol anhydrous, 99.8%; methanol hypergrade for LC-MS, LiChrosolv^®^; 2-propanol hypergrade for LC-MS, LiChrosolv^®^; n-hexane hypergrade for LC-MS, LiChrosolv^®^; ammonium acetate for LC-MS, LiChropur^®^; acetic acid, 100%, for LC-MS, LiChropur^®^; glacial acetic acid, 99.5%; menaquinone-7 (MK-7) United States Pharmacopeia (USP) reference standard; and vitamin MK-7 100 μg mL^−1^ in acetonitrile, certified reference material (check standard) were purchased from SIGMA Aldrich (Gliwice, Poland).

### 2.3. Preparing a Sample—Hard Tablets

The hard tablets were prepared in accordance with the methodology described by Szterk et al. [2].

### 2.4. Preparing a Sample—Gelatine Capsules

After establishing the mean mass of a gelatin capsule, 10 gelatin capsules were cut open, and their contents were squeezed out into a beaker. Approximately 2.5 g ± 0.1 g of the oil fraction was weighed into a 5-mL measuring flask and dissolved in a 1:1 *v*/*v* mixture of THF:methanol, which was added until the flask capacity was reached. The prepared sample was then subjected to fractioning using a semi-preparative chromatographic column.

### 2.5. Fractioning

The fractioning was conducted using an Accela Model 430 bar liquid chromatograph (Thermo Scientific, Waltham, MA, USA) equipped with a four-component pump, making it possible to mix four different ingredients of the mobile phase under low pressure; an auto-sampler, which allowed the cooling of the samples to 5 °C ± 0.05 °C and the feeding of a 300-µL sample into the chromatographic column; a thermostat for the column; and a diode array detector (DAD, λ = 185–1100 nm). The fractions were collected manually. For the fractioning, a COSMOSIL Cholester (Kyoto, Japan) 10 × 250 mm semi-preparative chromatographic column with a 5-µm grain diameter (code: 05979-31) was used. The column temperature was 20 °C ± 0.05 °C. The separation was conducted isocratically under the condition of a constant flow of the mobile phase through the column, amounting to 4.5 mL min^−1^, and the analysis time was 150 min. The mobile phase used for fractioning the menaquinones was obtained by mixing 70% of the A phase and 30% of the B phase under low pressure. During the chromatographic separation, the percentage shares of the phases were not changed.

Phase A, glacial acetic acid:H_2_O:methanol (1:1:8, *v*/*v*/*v*); Phase B, glacial acetic acid:isopropanol:hexane (1:5:2.5, *v*/*v*/*v* Then, 300 µL of the previously prepared oil sample was injected on the chromatographic column.

**Figure 2:**
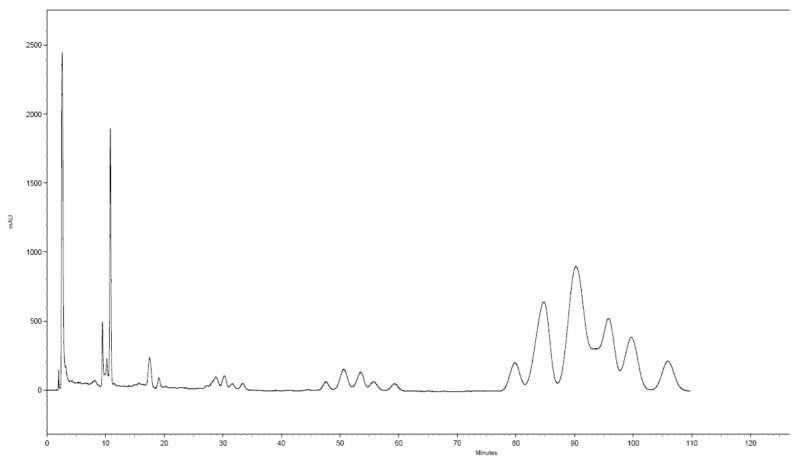
Example of a chromatogram showing the fractionation of menaquinones from oil samples. COSMOSIL Cholester 10 mm × 250 mm semi-preparative chromatographic column with a 5-µm grain diameter.

Figure 2 presents an example of a chromatogram, showing the fractioning of menaquinones using the described method. Two fractions were collected: the menaquinones-6 fraction at 45–62 min and the menaquinones-7 fraction at 76–110 min. Both fractions were combined in a round-bottom flask with a cut and evaporated until dry in a vacuum evaporator at 50 °C ± 1.0 °C. The dry remnant was dissolved in 1 mL of a 3:7 *v*/*v* THF:methanol mixture and analysed.

### 2.6. Chromatographic Analysis, DAD, and HRMS-QTOF

The menaquinone content analysis was conducted in accordance with the methodology described previously by Szterk et al. [2], with one modification: the chromatographic separation was conducted isocratically with a constant 30% of the B phase, which made it possible to better separate the *cis*/*trans* menaquinone isomers.

### 2.7. Method Validation

Quantitative analysis was performed by the standard-addition method. In this way, besides estimating the amount of the analytes occurring in the different dietary supplements, it was possible to also evaluate the sensitivity (LOD and limit of quantification, LOQ) and linear dynamic range in the various matrices. The recoveries, matrix effect, linearity, accuracy (repeatability and reproducibility), LOD, and LOQ were calculated after determining the levels of the all-*trans* MK-7 in the specific food.

#### 2.7.1. Preparation of Stock, Spiking Solutions, and LOD and LOQ Calculation

A primary stock solution of all-*trans* MK-7 (5.0 mg mL^−1^) was prepared in a methanol:THF (7:3 *v*/*v*) solution. Analyte spiking solutions (10 and 20 µg mL^−1^) were prepared with the intermediate mixed solution of the analyte, and all these solutions were stored at −20 °C and protected from light. Twelve calibration curve points (0.2, 0.4, 0.8, 1.6, 3.2, 6.4, 12.8, 25.6, 51.2, 102.4, 204.8, and 250.0 µg mL^−1^) were prepared by spiking the matrix after evaporation and sample extraction. The LOD and LOQ were then calculated. The LOD was defined as three times the standard deviation of the blank, and the LOQ was defined as ten times the standard deviation of the blank. The mean LOD and LOQ were calculated from each of the ten studied samples (*n* = 10), independent of the applied detectors.

#### 2.7.2. Recovery

The recovery of the analyte (all-*trans* MK-7) was studied using the standard addition method. The recovery was analysed at three different levels of enrichment (10 µg, 20 µg, and 30 µg per mL of final solution); such an approach was necessary due to the different masses of the studied tablets and capsules. The recovery was independently assessed for each of the supplements studied and detectors used (DAD and quadrupole time of flight, QTOF). The global recovery for each level of enrichment (mean of all the studied supplements, *n* = 40) and for each detector was calculated.

#### 2.7.3. Matrix Effect (ME)

The ME was calculated as 100% − {[peak area of MK-7 in the presence of matrix (post extracted sample)/mean peak area of MK-7 in the absence of matrix (all-*trans* MK-7 in methanol:THF solution)] × 100%}. The mean ME was calculated for *n* = 40, independent of the applied detectors.

#### 2.7.4. Precision and Accuracy

Precision and accuracy were evaluated for intraday and inter-day parameters. The intraday precision was calculated as the coefficient of variation percentage (% CV) from the mean value of the quality control samples (all-*trans* MK-7 USP Reference Standard, *n* = 10, and concentration of 20 µg mL^−1^) for an individual using the same equipment under the same conditions and over a short period of time (one day). The inter-day precision was calculated as the % CV from the mean value of the quality control samples (all-*trans* MK-7 USP Reference Standard, *n* = 10, and concentration of 20 µg mL^−1^) for three individuals using the same equipment under the same conditions and over a longer period of time (3 days). The accuracy of this analytic method was assessed as the percent relative error (100 × (found − added)/added). The analytical standard, 20 µg of all-*trans* MK-7 per sample for a final concentration of 20 µg mL^−1^, was always added after the entire sample preparation procedure was completed. The accuracy was measured for one randomly-selected dietary supplement, *n* = 10. The precision and accuracy were performed according to Yilmaz et al. [29].

## 3. Results

A stereoselective, semi-preparative, analytical chromatographic column filled with chemically modified silica containing approximately 20% substituted cholesteryl groups was used to determine the *cis*/*trans* menaquinone isomers in the studied dietary supplements. The quantitative determination of the geometrical isomers of vitamin K_2_ was conducted using DAD. Identification of the *cis*/*trans* isomers was conducted using QTOF.

**Figure 3:**
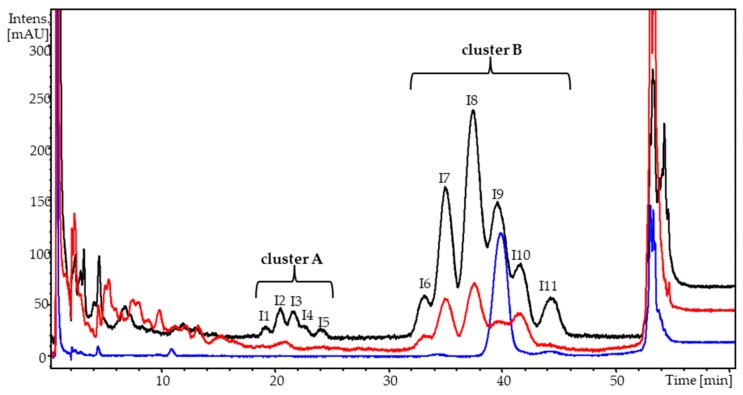
Chromatographic separation of analytical standard of MK-7 vitamin (blue line), a dietary supplement in the form of an oil capsule—MK-7 dissolved in a vegetable oil (red line), and a dietary supplement in the form of a hard tablet (black line). COSMOSIL cholester column (2.0 mm × 150 mm), with a 3.0 µm grain diameter. Retention time for analysed isomers in min.: I1—19.50; I2—20.71; I3—21.90; I4—22.75; I5—22.94; I6—33.42; I7—35.21; I8—37.60; I9—39.92; I10—41.63; I11—44.62.

**Figure 4:**
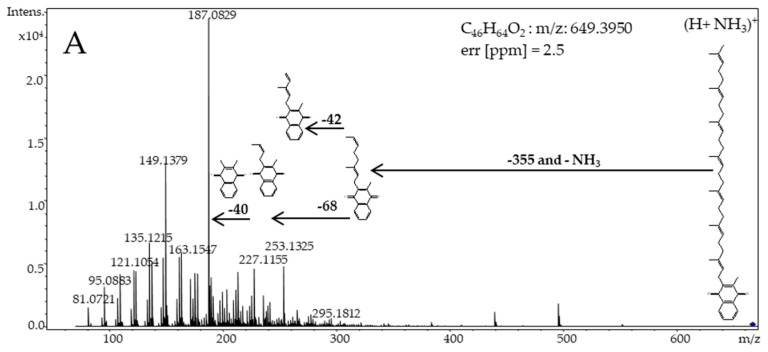

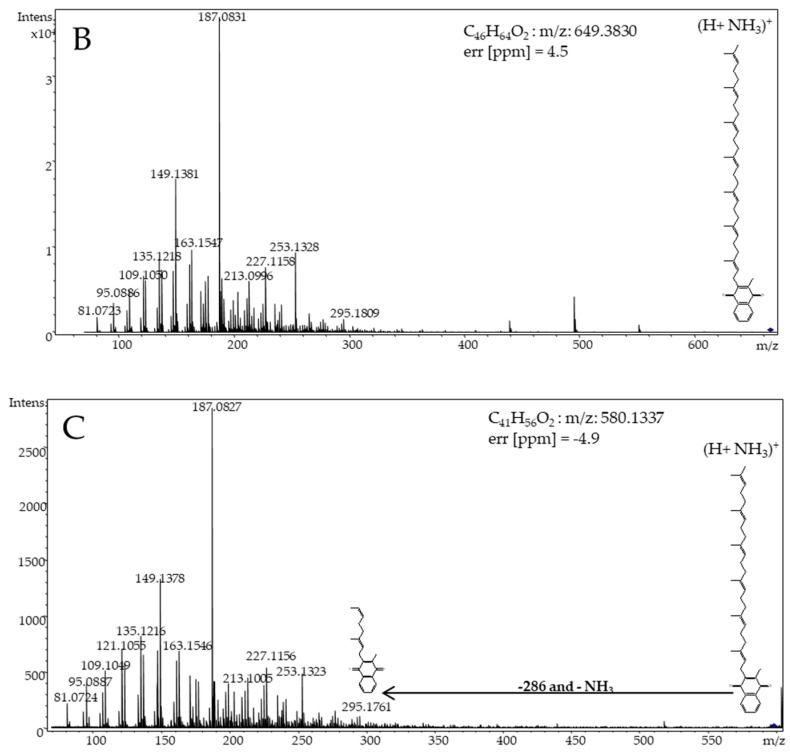
Fragmentary spectrum (**A**): standard of MK-7 vitamin, and two group peaks within the range (**B**): 31–43 min, (**C**): 18–25 min.

Figure 3 presents examples of the chromatograms for the MK-7 analytical standard and the dietary supplements, in the forms of gelatin capsules containing vitamin K_2_ dissolved in vegetable oil and hard tablets, in which the impurities of menaquinone-7 were identified. Two clearly visible concentrated peaks appear in Figure 3within the ranges of 18–25 min (cluster A) and 31–43 min (cluster B), for both the oil capsules and hard tablets. Unknown substances were identified using a high-resolution mass spectrometer combined with a highly efficient liquid chromatograph (HRMS-QTOF). Figure 4 shows a fragmentary spectrum of an all-*trans* MK-7 analytical standard and the fragmentary spectra of the two chromatographically separated groups of peaks visible in Figure 3 (clusters A and B). All the spectra of unknown peaks are presented in Figure S1, Supplementary Materials.

Compound I9 (Figure 3) is a quasi-molecular ion formed as the result of the addition of NH_4_^+^ to 649.00 (M + H + NH_3_)^+^ and an all-*trans* MK-7 vitamin having *m*/*z* = 667.00. The retention time of compound I9 and its fragmentary spectra were identical to the MK-7 analytical standard (Figure 3 and Figure 4A,B). The remaining recorded spectra of the unknown substances: I6, I7, I8, I10, and I11 (Figure S1, Supplementary Materials) did not differ statistically from the spectrum of the all-*trans* MK-7. 

Moreover, these substances ionised in exactly the same way as did the all-*trans* MK-7 (M + H + NH_3_)^+^, which caused the formation of an adduct, a quasi-molecular ion of a compound having a theoretical mass of 649.00 g mol^−1^, with *m*/*z* = 667.00. Because of the fragmentation of the ion with *m*/*z* = 667.00, the detachment of ammonium occurs, causing in each case the formation of a quasi-molecular ion of the compound C_46_H_64_O_2_ with an actual *m*/*z* = 649.5. For all the studied substances visible in Figure 3, cluster B, and Figure S1, Supplementary Materials, the mass measurement error was less than 5 ppm, and the isotope profile (mSigma) had low values, resulting in a 99.999% match for the molecular formula.

A fragmentary spectrum of compounds I1 through I5, visible in Figure 3 (cluster A), is presented in Figure 4C. All the compounds I1 through I5 had the same fragmentary spectrum (Figure S1, Supplementary Materials). Based on analysis of this spectrum and a quasi-molecular ion (*m*/*z* = 579.00), it was ascertained that these were the *cis*/*trans* isomers menaquinone-6, having an *m*/*z* = 580.00 g/mol. A quasi-molecular ion, formed as the result of the addition of NH4^+^ to 580.00 (M + H + NH_3_)^+^, had *m*/*z* = 597.00. As the result of the fragmentation of an ion having an *m*/*z* = 597.00, the detachment of ammonium rest occurred, which caused, in each case, the formation of a quasi-molecular ion of a compound having the molecular formula C_41_H_56_O_2_ and an actual *m*/*z* = 581.5. For all the studied substances that are visible in Figure 2, cluster A, and Figure S1, Supplementary Materials, the mass measurement error was less than 5 ppm, and the isotope profile (mSigma) had low values, resulting in a 99.999% matching for the molecular formula. As additional confirmation, the recorded unknown peaks in both forms of the dietary supplements were the *cis*/*trans* isomers of menaquinone-6, demonstrating a fragmentation mechanism identical to menaquinone-7. The difference was that the quasi-molecular ion of the peaks visible in Figure 3, cluster A, was smaller by one prenyl unit, i.e., by 69 g mol^−1^. For that reason, as well, the first fragment visible in Figure 2C was formed after the detachment of a rest having *m*/*z* = 286 from menaquinone-6.

On the basis of the conducted research, it is possible to ascertain that substances I1 through I5, identified in both the oil capsule and hard tablet forms of the MK-7 dietary supplements, were quite likely the *cis*/*trans* isomers of menaquinone-6, while peaks I6 through I11 were quite likely the *cis*/*trans* menaquinone-7 isomers, which were identified for the first time ever in food. Unfortunately, with the application of HRMS, it was not possible to ascertain the locations of the bonds with the *cis* configuration on the isoprene chains of menaquinone-6 and menaquinone-7.

Table 1 presents the validation parameters for the method developed for quantifying the menaquinones dissolved in vegetable oil and enclosed in a soft gelatin capsule. A method for quantifying the *cis*/*trans* isomers previously developed by Szterk et al. [2], was applied for quantifying the impurities of MK-7 in the hard tablets. Table 2 presents the results for *cis*/*trans* MK-6 and MK-7 isomer content in the studied dietary supplement samples.

In Table 1, the LOD, LOQ, and linearity, are expressed in µg mL^−1^ However, a more practical and useful unit might be µg tablet^−1^ or µg capsule^−1^. However, because the tablet and capsule masses varied greatly (Table 2), it was decided to express all the units as µg mL^−1^_,_ which significantly eased the presentation and comparison of the results. Considering the sample preparation methodology, it would be easy to convert the results in Table 1 and express them as per tablet or per capsule. Based on the results shown in Table 1, the method developed was sufficiently sensitive in terms of quantifying the MK-7 in the dietary supplements. The sensitivity of the method (LOD and LOQ) depended on the detector used. 

The greatest sensitivity (lowest LOD and LOQ) was obtained using the QTOF. However, the linearity of the QTOF method was very low, which essentially disqualified this detector from quantification use. QTOF was the ideal tool for the identification or confirmation of the quantified substances.

The DAD was very suitable for quantifying the MK-7 in the dietary supplements. The LOD and LOQ for this detector were sufficient for quantifying the *cis*/*trans* forms of menaquinones in food. Moreover, this detector had a very broad range of linearity, from the LOQ to 250 µg mL^−1^. The recovery provided by this method did not depend statistically on the applied detector, resulting from the small matrix effect (ME < 5%), and ranged between 85% and 94.6%. The method developed was highly precise, which, in case of the DAD, amounted to an intraday precision of 0.8% and inter-day precision of 4.1%. All validation parameters studied were evaluated based on the all-*trans* MK-7 isomers. Unfortunately, no *cis*/*trans* forms of menaquinones are sold. However, the application of the DAD for quantification essentially eliminated the problem of the lack of standards for the *cis*/*trans* isomers because the signal of this detector depended only on the concentration and not significantly upon the spatial structure of the studied molecules. For that reason, as well, the method developed for quantifying the *cis*/*trans* menaquinones isomers with a DAD could be applied to research the quality of different MK-7 dietary supplements available on the pharmaceutical market. Even though all-*trans* menaquinone-6 is for sale, 5 mg of this compound costs approximately 12,750 USD (Toronto Research Chemicals, ON, Canada); thus, the quantification of the isoform menaquinone-6 was performed for the all-*trans* menaquinone-7.

The research performed gives rise to the conclusion (Table 2) that the composition of the dietary supplements with regard to the contents of MK-6 and MK-7 was extremely varied. The MK-7 content of the hard tablet supplements was significantly lower (between 22.7 and 87.7 µg per tablet) than that of the oil capsules (between 26 and 373.8 µg per capsule). The *cis*/*trans* isomers of menaquinone-6 and menaquinone-7 were not found in all the hard tablets or in all the oil capsules (Table 2). The *cis*/*trans* menaquinone isomers were found in a supplement which, as the producer declared, contained the vitamin from a natural natto extract, as well as in a supplement for which the producer did not declare the origin of the MK-7. It is interesting that, in the majority of the studied oil capsule dietary supplements, the MK-7 content was many times greater than that declared on the label. Such a trend was not established in case of the studied hard tablets.

## 4. Discussion

For the first time, Szterk et al. [2] determined the *cis*/*trans* menaquinone-7 isomer content in dietary supplements in the form of hard tablets. There are also supplements on the market in the form of oil capsules, wherein this vitamin is dissolved in different vegetable oils. Thus far, the oil supplements have not been studied in terms of their MK-7 content or isomeric forms of menaquinones. Analysing the content of these small, fat-soluble vitamins presents quite an analytical problem, connected with the presence of triacylglycerols, which are responsible for a very strong matrix effect that significantly limits the ability to precisely identify and quantify the small quantities of menaquinones. In order to quantify the menaquinones in the studied dietary supplements, attempts were made to use oil sample purification methods, with the application of extraction to the solid phase, using small columns filled with a silica gel in accordance with Booth et al. [30,31], and Ferreira et al. [32]. Unfortunately, the methods described in the global literature failed to provide a good result due to a very low recovery of menaquinone-7 from the studied matrices. For that reason, to quantify the *cis*/*trans* MK-6 isomers and MK-7, the method of fractioning with the application of a semi-preparative column containing a cholesteryl group was used, making it possible to separate the menaquinones from the triacylglycerol fraction. There were attempts to use a standard semi-preparative C18 column, however, together with the fractions of menaquinone-6 and -7, the triacylglycerol was also rinsed out, which made it impossible to precisely separate the matrix components. An initially fractioned sample that did not contain a triacylglycerol fraction was analysed using an analytical version of the same chromatographic column containing a cholesteryl group, which had a much higher chromatographic efficiency, in accordance with the methodology described by Szterk et al. [2]. The method developed for preparing the oil samples for the quantification of their different menaquinones provided high levels of recovery, was not very laborious, and could possibly be fully automated by equipping a liquid chromatograph with a fraction collector.

Based on the analysis of the mass spectra obtained (Figure 4 and Figure S1, Supplementary Materials), the separated substances are shown in Figure 2, cluster A, to be the *cis*/*trans* menaquinone-6 isomers (Compounds I1 through I5) and *cis*/*trans* menaquinone-7 isomers (cluster B). All these compounds ionised, producing quasi-molecular ions having theoretical *m*/*z* values of 597.00 (menaquinone-6) and 667.00 (menaquinone-7), being adducts of NH_4_^+^ to the theoretical masses, 580.00 and 649.00, respectively. The ionisation mechanism may be recorded in the form of (M + H + NH_3_)^+^. Forming an adduct with NH_4_^+^ relates to the fact that, in the mobile phases used for the chromatographic separation, ammonium acetate was used as a phase modifier. Forming such an adduct is typical for the applied phases [2,33,34]. The adducts of NH_4_^+^ are specific because, as the result of the fragmentation of such an ion, (M + H)^+^ and a neutral NH_3_ form. The (M + H)^+^ ion undergoes further fragmentation, producing a typical spectrum of menaquinones (Figure 4A–C and Figure S1, Supplementary Materials). The research performed with the application of HRMS (QTOF) made it impossible to ascertain in which place of an isoprenoid chain a *cis* bond is located. Both *cis* and *trans* isomers undergo fragmentation identically [28,35]. From the point of view of the dietary supplement quality and biological function, the identification of the all-*trans* MK-7 isomer and the *cis*/*trans* isomers MK-6 and MK-7 is quite sufficient. To determine the location of the *cis* bonds in an isoprenoid chain of menaquinones, it is necessary to conduct the fractioning of the substance, as shown in Figure 1, and then determine the chemical structure using nuclear magnetic resonance (NMR).

Researching the *cis*/*trans* isomers of MK-6 and MK-7 is very important from a biological point of view because only the *trans* forms of vitamins K_1_ and K_2_ manifest biological activity. The *cis* or *cis*/*trans* forms of menaquinones do not manifest such activity or manifest it at a very low level [5,9,28,36], this has been proven in the case of *cis* vitamin K_1_ (Lowenthal and Rivera [25]) and mixtures of the *cis*/*trans* MK-6 isomers [37]. Based on these publications, it can be concluded that the *cis* or *cis/trans* isomers are chemical impurities of MK-7 and MK-6. Geometrical isomerization of the isoprenoid units in menaquinones is possible in a few cases. The *cis* isomers are formed during the chemical synthesis of this vitamin [19,20,21,22], and during a poorly conducted technological process, the objective of which was to obtain different menaquinone preparations. It primarily involves the impact of light, in particular, UV radiation, which causes the geometrical isomerization of the isoprenoid units in the menaquinone chains [28]. Next, oxidation catalysed by high temperature or the action of radicals causes the formation of epoxides and *cis* isomers in different locations on the isoprenoid chains of the menaquinones [38]. This process may occur during the manufacture of MK-7 microcapsules, which are the initial raw material for the production of hard tablets or capsules. It may be presumed that the geometrical isomerization of all-*trans* MK-7 and MK-6 may occur as the result of the process of autooxidation during the storage of the dietary supplements, which are in contact with atmospheric oxygen, light, and elevated temperatures. Unfortunately, there are no relevant data in the literature. However, as the result of the research performed, we established the occurrence of the different *cis*/*trans* MK-6 isomers and MK-7. Moreover, in some of the supplements, the sum of the *cis*/*trans* menaquinone isomers was significantly greater than the content of the all-*trans* form of MK-7 (Table 2, Figure 3). The occurrence of a large quantity of *cis*/*trans* isomers in supplements is likely connected with the use of preparations containing synthetic MK-7 that was not subjected to an additional purification process (alternatively, the process was not very effective). The producers of certain supplements declared that the vitamin contained in them was from natural sources (natto extract). However, natto does not contain the *cis*/*trans* form of MK-7 or a different form of vitamin K_2_ but only the all-*trans*MK-7 isomer [2,15,18,23,24]. In turn, during the chemical synthesis of MK-7, the *cis*/*trans* isomers are formed in quantities relating to the quantities of the pure all-*trans* form by ratios of 1:3 or 1:2, or not at all, depending the method of synthesis; in addition, it is well-known that there are other forms vitamin K_2_, such as MK-11, MK-6, MK-5, and more [19,20,21,22]. In the analysed samples, large quantities of the *cis*/*trans* isomers were discovered, which would indicate the use of synthetic MK-7 and were likely a negative effect of the technological processes of obtaining the menaquinone preparations and, ultimately, a tablet/capsule ready for administration. Revealing the presence of the *cis*/*trans* form of MK-6 further proves that synthetic MK-7 preparations were used in the studied dietary supplements. Moreover, in these types of supplements, it can also be expected that there are different forms of vitamin K_2_, having an ever-smaller number of prenyl fragments. Analysing the literature concerning both chemical and biosynthetic methods of obtaining MK-7 preparations reveals that all the methods are based upon attaching further prenyl fragments to the 2-methyl-1,4-naphthoquinone rest, resulting in the MK-7 form (with MK-1 through MK-6 obtained in the progress) [18,22]. Depending on the methods of synthesis/biosynthesis and purification of the post-reaction mixture, the ultimate preparation may contain both the *cis*/*trans* form of MK-7 and other forms of menaquinones, in particular, MK-6, also in *cis*/*trans* form. The form MK-6 is the penultimate stage of the synthesis/biosynthesis process, and it most likely cannot be completely converted into aMK-7 form, thus remaining in the final product [22]. It needs to be highlighted that, in preparations obtained by means of chemical or biochemical synthesis, the presence of forms of vitamin K_2_ with more than seven prenyl fragments can be detected, which results from the specific character of the method of synthesis/biosynthesis. Analysing the content and profile of the impurities of MK-7, mainly, the *cis*/*trans* menaquinone isomers having a number of prenyl fragments between 1 and 11, may be a very good approach for determining the method by which the MK-7 present, e.g., in dietary supplements, was obtained. It is also worth noting that this hypothesis requires conducting a lot of research worldwide in order to collect sufficient evidence. At the moment, it is known that dietary supplements present on the market contain different forms of menaquinones, in particular MK-7, MK-6, and a number of *cis*/*trans* menaquinone isomers.

Apart from the presence of the *cis*/*trans* menaquinone isomers, an important problem was revealed in terms of the declared MK-7 content. In only a few supplements was the declared MK-7 content similar to the measured one. In the case of the oil capsule supplements, this content was a few times higher than the declared one, which may be explained in two ways. One possibility is that the producers wanted the vitamin content at the expiration date be at least as high as the level declared on the label, so that made them increase the initial quantity of the vitamin. Vitamin K_2_ is fairly unstable in terms of oxidation, causing its content to decrease while being stored [39]. Because producers declare long usability periods for their MK-7 dietary supplements (approximately 12 months, and up to 24 months), they include an excessive content of this vitamin. Another possible reason for the significantly higher concentrations of MK-7 in the oil capsule supplements may be the analytical problems connected with determining its content precisely in oil fraction. Measuring the MK-7 content in oil samples without prior laboratory preparation that makes it possible to eliminate the triacylglycerol fraction causes a significantly strong matrix effect, and, consequently, lowers the measured results. With lowered analytical results as the basis, mixing the oil concentrate with the oil used to produce the soft oil capsules will result in elevatedMK-7 content.

The results of the research performed lead to the formulation of several conclusions. The producers of dietary supplements take advantage of the lack of regulations regarding the quality of dietary supplements. In accordance with the laws currently in force in Europe and elsewhere, dietary supplements are considered to be in the category of food, which means that they only have to be safe. There are no effective regulations or solutions that would force the producers to conduct thorough quality control, such as conducted in the case of producing medicinal products. The result is that the actual vitamin content in the final product is different from that declared by the producer. This is affected by the dishonesty of producers, errors in the supplement production process (errors in recipes), and oxidising decomposition of the vitamin during production and transport, as well as storage of the supplements in shops, pharmacies, or homes. Ascertaining a high *cis*/*trans* menaquinone isomer content related to the all-*trans* MK-7 requires the verification of the biological activity and toxicity of the *cis*/*trans* form menaquinones present in different vitamin K_2_ supplements.

## 5. Concluding Remarks

A new method of quantifying menaquinones, including the *cis*/*trans* isomers, in dietary supplements sold in the form of soft capsules, in which the MK-7 is dissolved in different vegetable oils, was developed. The chromatographic method developed, using DAD and QTOF detection, made it possible to separate and quantify 11 different *cis*/*trans* MK-6, MK-7 isomers and the all-*trans* isomer of MK-7. The developed and validated method was used to study the content and different isomeric forms of menaquinones in dietary supplements produced in different countries and available on the market. The research revealed significant diversity in the MK-7 content in the studied hard tablet dietary supplements, which had concentrations below the declared level. In the case of the oil capsule supplements, the MK-7 vitamin content was significantly higher than declared. Moreover, it was revealed that there were large quantities of the *cis*/*trans* vitamin isomers (menaquinone-6 and -7), which most likely do not have vitamin K activity or it is significantly lower. Different MK-7 contents in the studied dietary supplements and the occurrence of large quantities of the *cis*/*trans* isomers may indicate the dishonesty of producers who declare that they use natural natto extracts for producing their supplements because such extracts do not contain *cis* isomers of this vitamin. It may be presumed that, actually, they used cheap preparations of vitamin K_2_ obtained by means of chemical synthesis, and for that purpose applied production technologies in which the *cis*/*trans* isomers were formed, or the sold product underwent chemical transformation (e.g., oxidation) while stored, causing the transformations of the all-*trans* MK-7 into the *cis*/*trans* isomers. For the first time, an attempt was made to research MK-7 dietary supplements, it is necessary to emphasize the significant problem of their quality resulting from the lack of appropriate requirements and controls.

## Figures and Tables

**Table 1:** Validation parameters of all *trans* MK-7 in oil dietary supplements.

	DAD Detector	QTOF Detector (SIM, *m*/*z* = 666.5 ± 0.5 *m*/*z*)
LOD (ng mL^−1^)	290.3	200.9
% CV	9.1	9.8
LOQ (ng mL^−1^)	957.9	603.7
% CV	0.3	9.7
Recovery:		
Spiked level 1: 10 µg mL^−1^ (%)	85.6	81.4
SD	1.7	6.1
Spiked level 2: 20 µg mL^−1^ (%)	89.3	90.5
SD	2.3	6.2
Spiked level 3: 30 µg mL^−1^ (%)	93.8	94.6
SD	0.9	4.7
Matrix effect (ME) (%)	<5	<5
SD	-	-
Linear (range in µg mL^−1^ and R^2^)	0.9–250 µg mL^−1^ R^2^ = 0.997	Very low, for example: 2–4 µg mL^−1^ R^2^ = 0.991, but for 2–20 µg mL^−1^ R^2^ = 0.860
Precision and accuracy:		
Intra-day precision in % CV	0.8	6.1
Intra-day accuracy	2.9	5.8
Inter-day precision in % CV	4.1	11.3
Inter-day accuracy	−2.9	−5.0

**Table 2:** All *trans* MK-7 and *cis*/*trans* isomers of MK-6 and MK-7 contents in dietary supplements obtained from local markets, *n* = 10.

	Sample	Average Weight of Pill (mg)	SD	Declared Amount of MK-7 Vitamin by Producer (µg pill^−1^)	all *trans* MK-7 (µg pill^−1^)	SD	Sum of *cis/trans* Isomers of Vitamin MK-7 (µg pill^−1^)	SD	Sum of *cis/trans* Isomers of Vitamin MK-6 (µg pill^−1^)	SD
Hard pills	Switzerland producer	394.2	2.9	75 (from natto)	80.7	11.0	218.7	28.5	16.9	2.3
	British producer 1	548.2	5.1	100 (from natto)	87.7	3.4	<LOD	-	<LOD	-
	British producer 2	300.8	6.7	100 (no data)	22.7	0.8	<LOD	-	<LOD	-
	Polish producer	232.2	2.2	75 (no data)	42.5	1.6	<LOD	-	<LOD	-
Soft pills (gelatine pills)	Polish producer 1	427.8	25.7	75 (no data)	26.0	0.4	70.9	1.1	5.5	0.1
	Polish producer 2	425.5	23.8	50 (from natto)	223.8	35.6	<LOD	-	<LOD	-
	Polish producer 3	972.9	15.0	50 (from natto)	224.8	23.4	<LOD	-	<LOD	-
	Polish producer 4	431.9	11.4	75 (from natto)	373.8	17.7	<LOD	-	<LOD	-

## Supplementary Materials

The following are available online. Figure S1: title: supplementary materials, chromatograms of two cluster peak represented for menaquinone-6 (I1-I5) and menaquinone-7 (I6-I11). QTOF MS/MS spectrum of each identified peaks from I1 to I11.
