# Supplementary material for: Analysis of Menaquinone-7 Content and Impurities in Oil and Non-Oil Dietary Supplements
Source: Molecules. 2018 May 1;23(5):1056. doi: 10.3390/molecules23051056 (PMC6102598; doi:10.3390/molecules23051056)
Supplement: Supplementary file 1 [file molecules-23-01056-s001.zip › molecules-288051-SI.pdf]

Figure S1 supplementary materials, chromatograms of two cluster peak represented for menaquinone-6 (I1-I5) and menaquinone-7 (I6-I11). Below was presented QTOF MS/MS spectrum of each identified peaks from I1 to I11

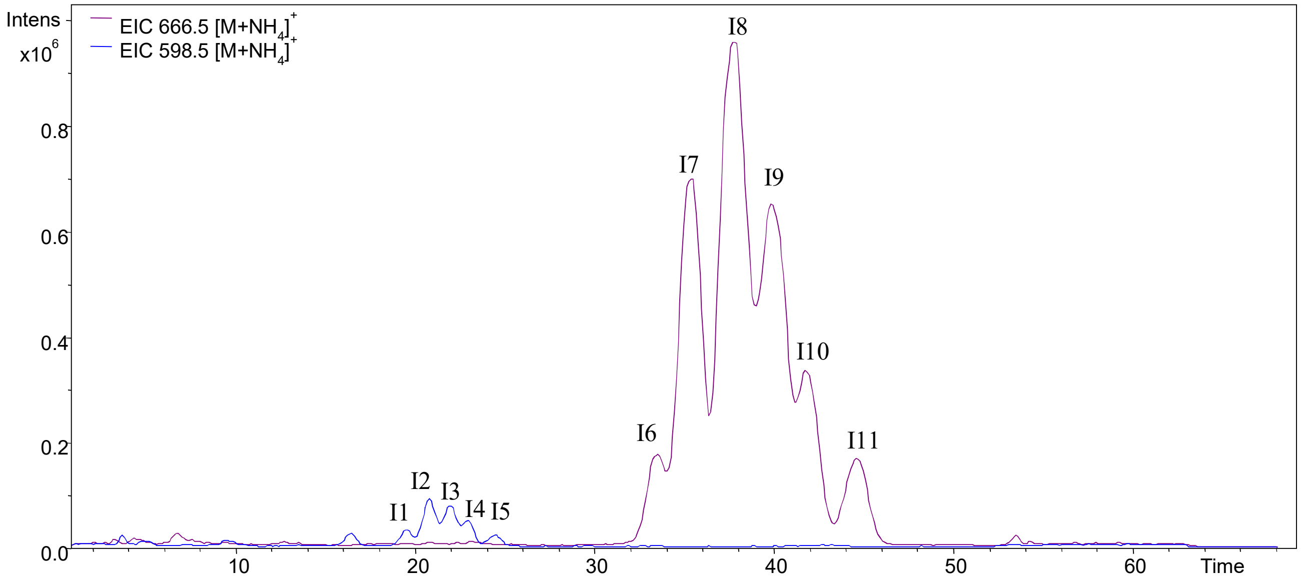

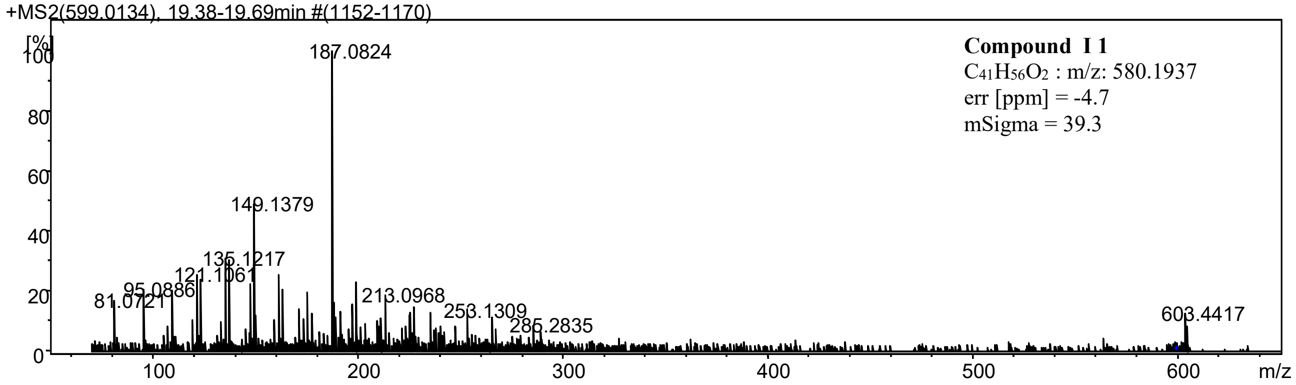

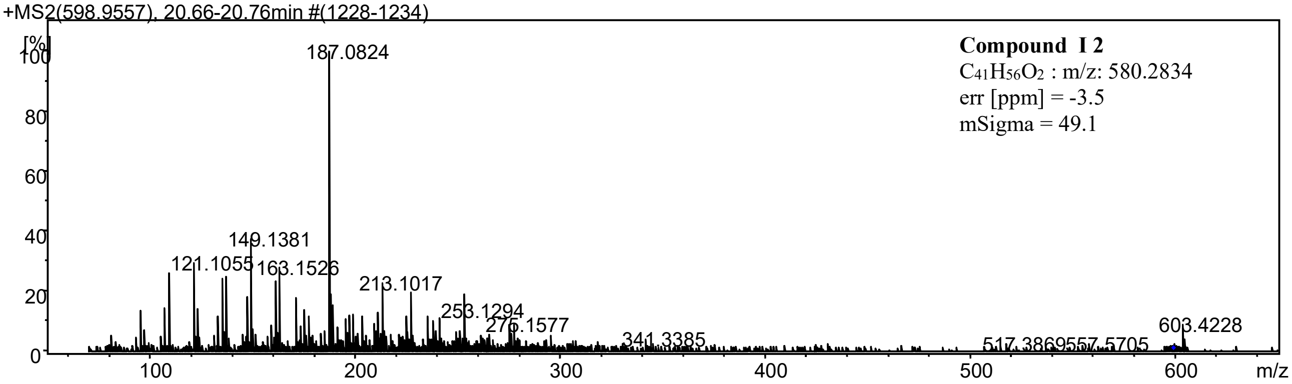

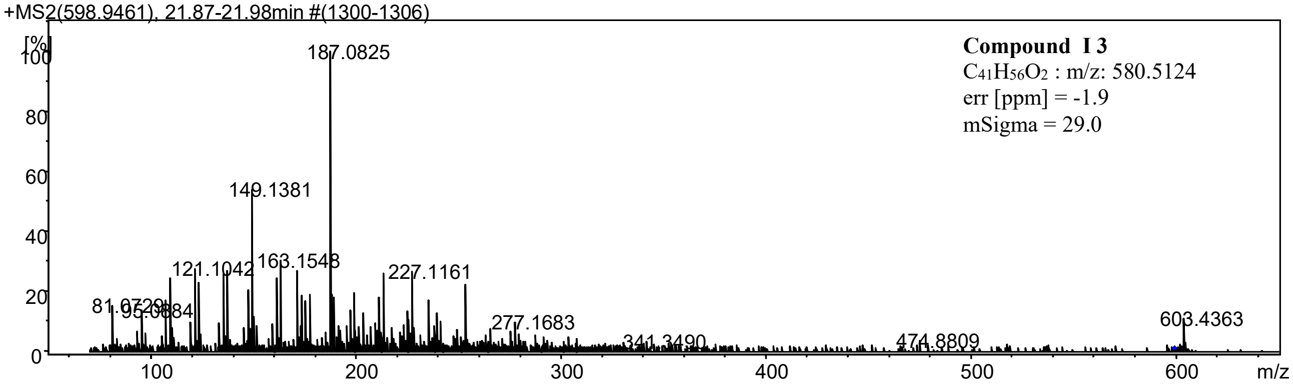
+MS2(598.9461), 21.87-21.98min #(1300-1306)
Compound I 3
C41H56O2 : m/z: 580.5124
err [ppm] = -1.9
mSigma = 29.0
187.0825
149.1381
121.1042
163.1548
227.1161
81.0729
95.0884
277.1683
341.3490
474.8809
603.4363
[%]
m/z

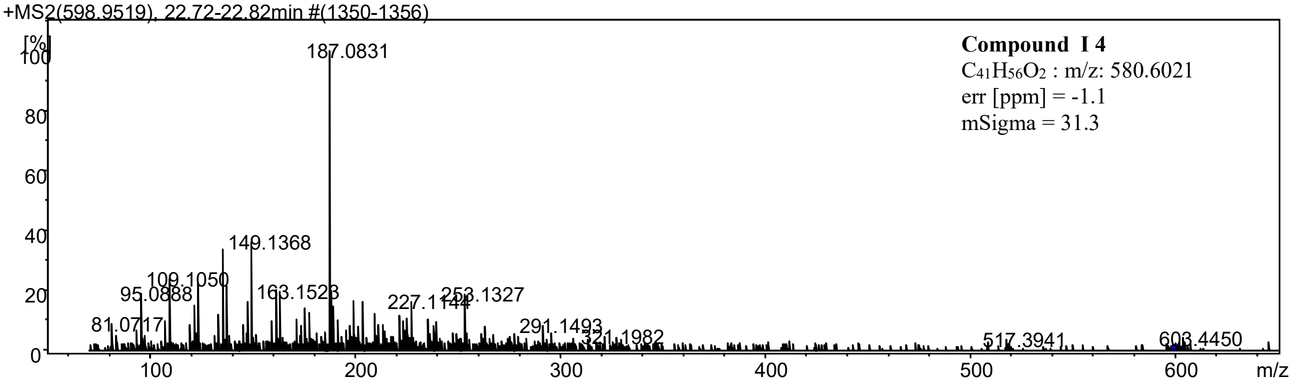
+MS2(598.9519), 22.72-22.82min #(1350-1356)
Compound I 4
C41H56O2 : m/z: 580.6021
err [ppm] = -1.1
mSigma = 31.3
187.0831
149.1368
109.1050
95.0888
163.1523
227.1144
253.1327
81.0717
291.1493
321.1982
517.3941
603.4450
[%]
m/z

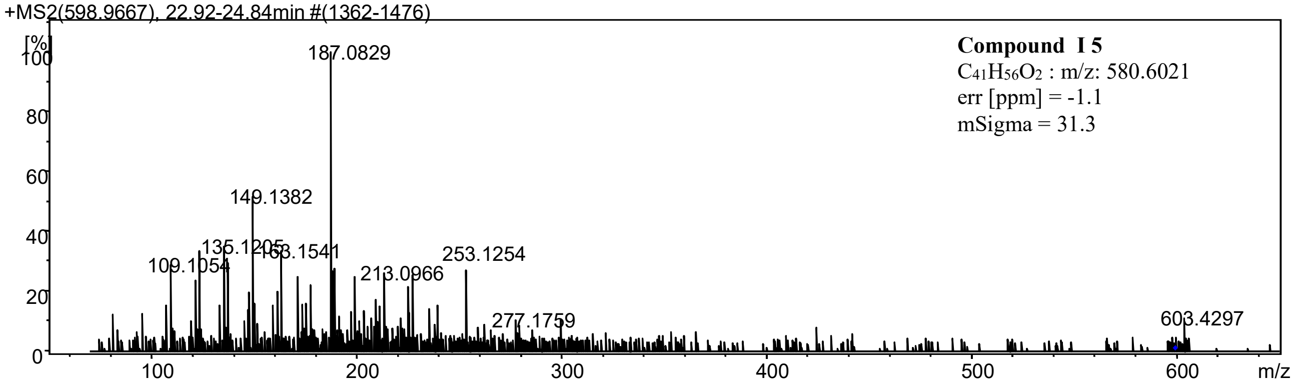
+MS2(598.9667), 22.92-24.84min #(1362-1476)
Compound I 5
C41H56O2 : m/z: 580.6021
err [ppm] = -1.1
mSigma = 31.3
187.0829
149.1382
135.1205
163.1541
109.1054
253.1254
213.0966
277.1759
603.4297
[%]
m/z

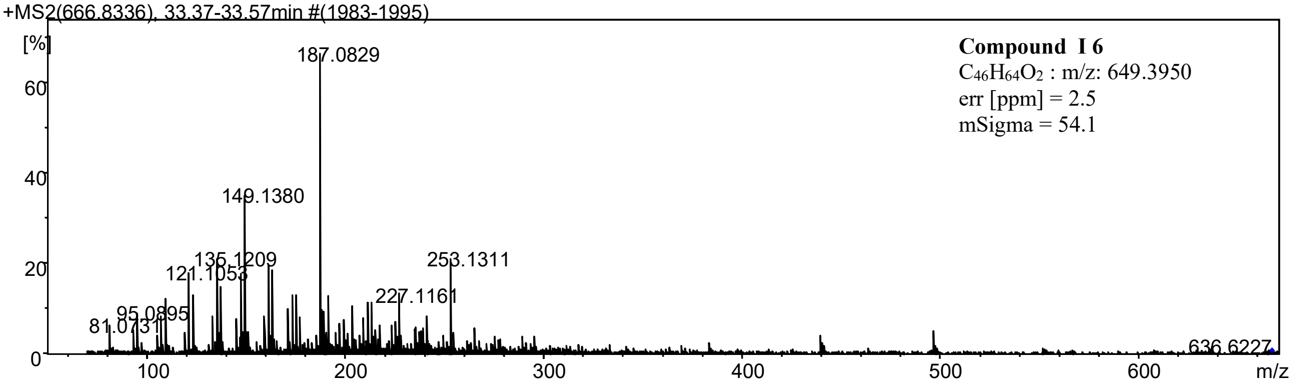
+MS2(666.8336), 33.37-33.57min #(1983-1995)
Compound I 6
C46H64O2 : m/z: 649.3950
err [ppm] = 2.5
mSigma = 54.1
187.0829
149.1380
135.1209
121.1053
253.1311
227.1161
95.0895
81.0731
636.6227
[%]
m/z

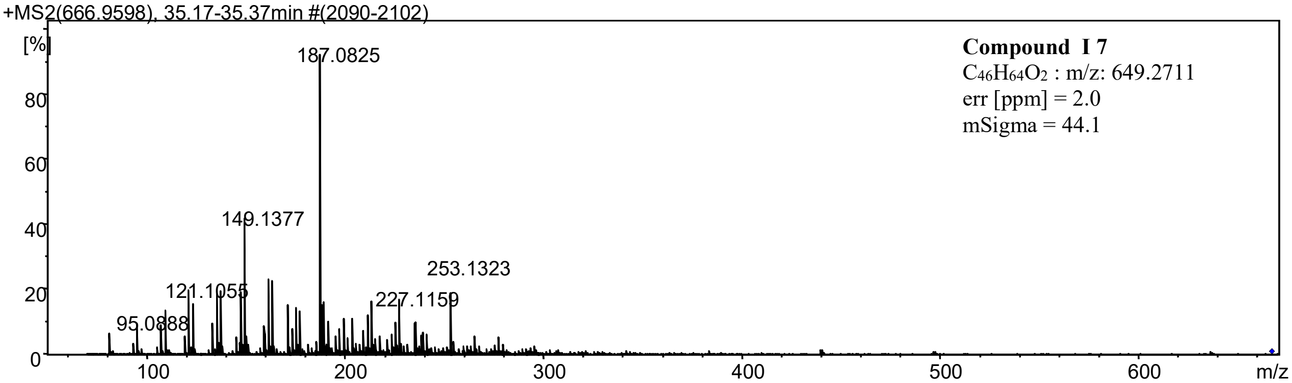
+MS2(666.9598), 35.17-35.37min #(2090-2102)
[%]
Compound I 7
C46H64O2 : m/z: 649.2711
err [ppm] = 2.0
mSigma = 44.1
187.0825
149.1377
253.1323
121.1055
227.1159
95.0888
m/z

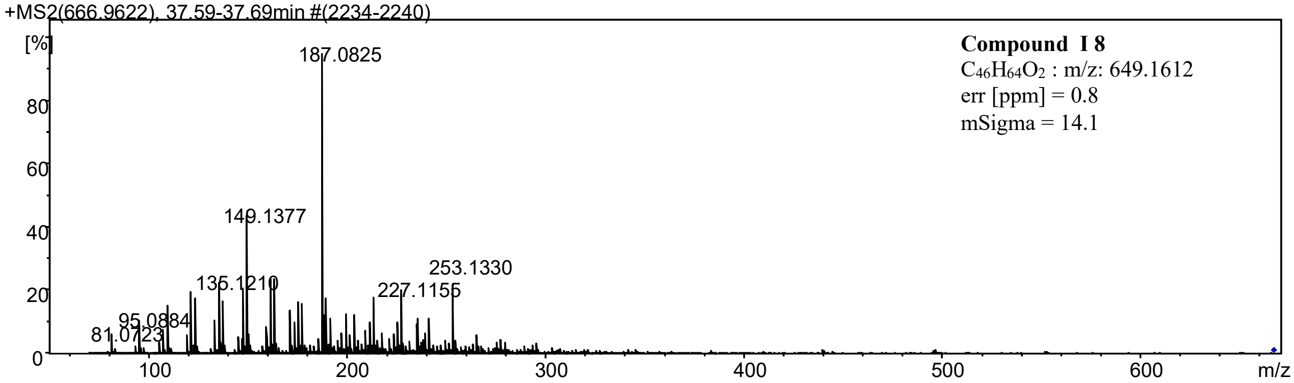
+MS2(666.9622), 37.59-37.69min #(2234-2240)
[%]
Compound I 8
C46H64O2 : m/z: 649.1612
err [ppm] = 0.8
mSigma = 14.1
187.0825
149.1377
253.1330
135.1210
227.1155
95.0884
81.0723
m/z

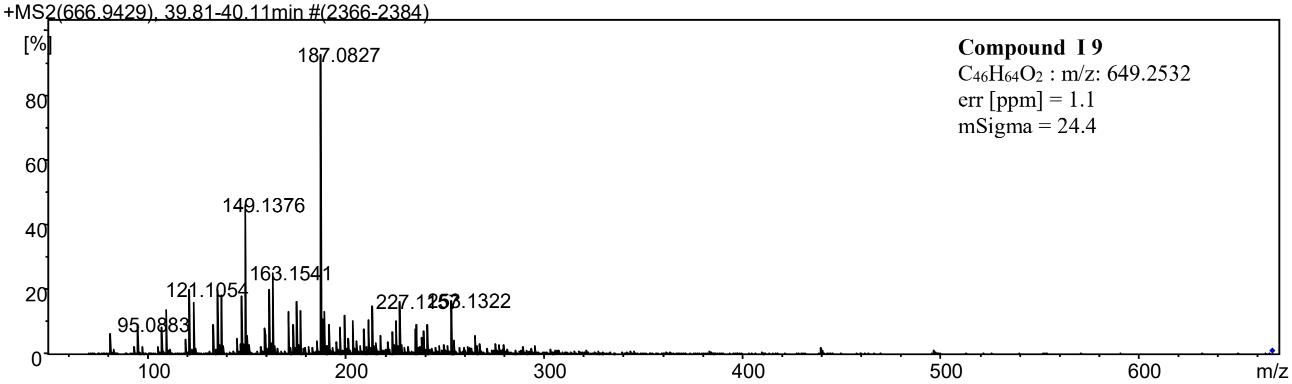
+MS2(666.9429), 39.81-40.11min #(2366-2384)
[%]
Compound I 9
C46H64O2 : m/z: 649.2532
err [ppm] = 1.1
mSigma = 24.4
187.0827
149.1376
163.1541
121.1054
253.1322
95.0883
m/z

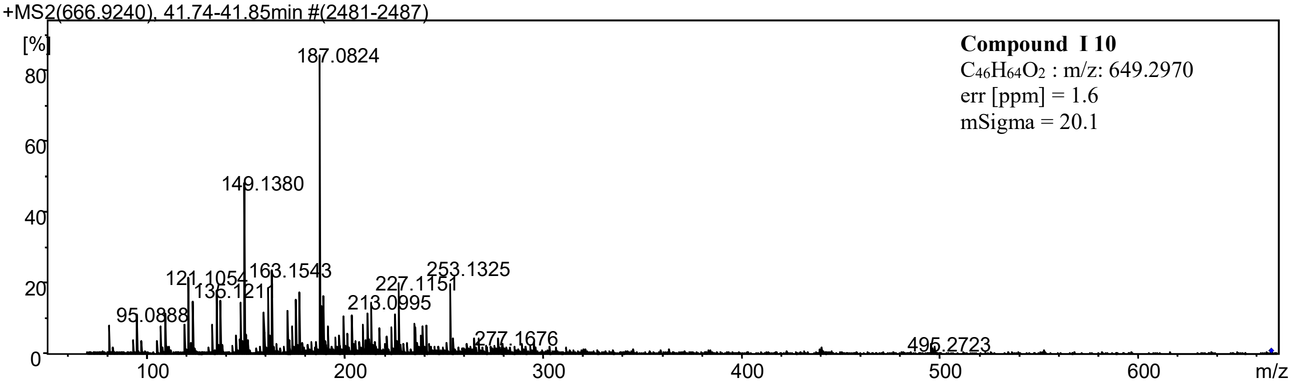
+MS2(666.9240), 41.74-41.85min #(2481-2487)
[%]
Compound I 10
C46H64O2 : m/z: 649.2970
err [ppm] = 1.6
mSigma = 20.1
187.0824
149.1380
121.1054
163.1543
253.1325
227.1151
135.1211
213.0995
95.0888
277.1676
495.2723
m/z

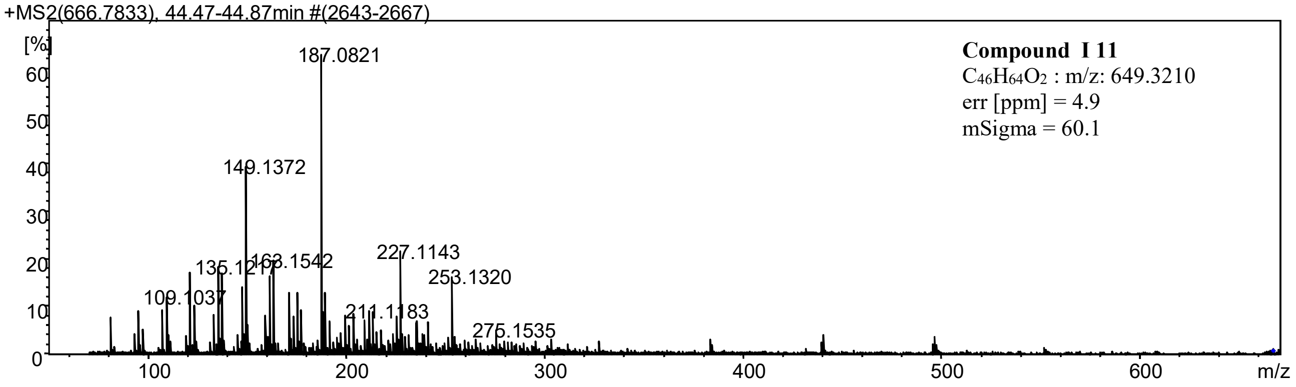
+MS2(666.7833), 44.47-44.87min #(2643-2667)
[%]
187.0821
149.1372
135.1217
163.1542
227.1143
253.1320
109.1037
211.1183
275.1535
m/z
Compound I 11
C46H64O2 : m/z: 649.3210
err [ppm] = 4.9
mSigma = 60.1
